# The Influence of Polyanions and Polycations on Bacteriophage Activity

**DOI:** 10.3390/polym13060914

**Published:** 2021-03-16

**Authors:** Egor V. Musin, Aleksandr L. Kim, Alexey V. Dubrovskii, Ekaterina B. Kudryashova, Elena V. Ariskina, Sergey A. Tikhonenko

**Affiliations:** 1 Institute of Theoretical and Experimental Biophysics, Russian Academy of Science, Institutskaya st. 3, 142290 Puschino, Russia; eglork@gmail.com (E.V.M.); kimerzent@gmail.com (A.L.K.); dav198@mail.ru (A.V.D.); 2 G. K. Skryabin Institute of Biochemistry and Physiology of Microorganisms, Federal Research Center, Pushchino Scientific Center for Biological Research, Russian Academy of Sciences, Prospect Nauki 5, 142290 Pushchino, Russia; katryn@ibpm.pushchino.ru (E.B.K.); lena@ibpm.pushchino.ru (E.V.A.)

**Keywords:** bacteriophages, polyelectrolytes, polyallylamine, polysteryne sulfonate, dextrane sulfate, polyarginine, *E. coli*, electrostatics

## Abstract

Phage therapy is a great alternative to antibiotic drugs, but it can’t effectively overcome the over-acidic medium of the stomach. We offer the use of polyelectrolyte microcapsules as a protective means of bacteriophage. It is necessary to understand the influence of polyelectrolytes on bacteriophage survival. The work studied the effect of polyanions and polycations on the coliprotetic bacteriophage’s viability. We have shown that polyallylamine decreased bacteriophage’s viability during increasing polyelectrolyte concentration and polyarginine had a lower inhibitory effect (then PAH) on the activity of the bacteriophage due to polyelectrolyte concentration from 0.05 to 5 mg/mL. It was shown that the inhibition of the bacteriophage by polyallylamine had an electrostatic nature and the use of high ionic strength prevented the formation of the PAH-protein capsid complex. Polystyrene sulfonate does not affect bacteriophage viability during increasing polyelectrolyte concentration from 0.05 mg/mL to 1 mg/mL. Polystyrene sulfonate decreases the viability of bacteriophage from 5 mg/mL of polyelectrolyte concentration. Dextran sulfate inhibits bacteriophage activity at 20–30%. Dextran inhibits bacteriophage activity by 80% at diapason concentration from 0.05 to 5 mg/mL and loses the inhibition effect from a concentration of 5 mg/mL.

## 1. Introduction

Antibiotics are widely used in modern medicine and agriculture to prevent and reduce the increase in the incidence of bacterial infections. Antibiotic-resistant pathogenic microorganisms appeared due to the active and poorly controlled use of antibiotics and, as a result, the problem is the effective treatment of diseases caused by these microorganisms [1]. According to the Centers for Disease Control and Prevention in the United States, the number of reported deaths due to antibiotic-resistant infections is 35,000 per year [2]. In this regard, the search for new methods of treatment, an alternative to antibiotic therapy is urgent [3].

Bacteriophages may be a new class of biological drug that has a specific bactericidal effect against specific types of microorganisms. The use of bacteriophages in medicine is based on their ability for highly specific affinity interactions with certain conservative surface structures of the corresponding bacterial cells. These cells have the biochemical and physiological potential for replication or integration of the genome of this bacteriophage [4,5], followed by lysis of the bacterial cell. Currently, phages are used as natural antimicrobial agents to combat bacterial infections in humans, animals, and crops [6,7,8,9,10]. Phage therapy has great prospects, the development of which is hindered by such factors as the difficulty of maintaining the viability of bacteriophages and the delivery of phages to the inflammation focus. Thus, it is necessary to develop effective delivery systems capable of protecting the bacteriophage from the external environment. One of the ways to create this system is to encapsulate bacteriophages in polyelectrolyte microcapsules.

The phage encapsulation literature focuses on gastrointestinal infections. The reason for encapsulation is the need to protect the phage from the acidic environment of the stomach, in which the free phage is inactive or its titer decreases [11]. For example, Colom et al. encapsulated bacteriophages in alginate microcapsules containing a CaCO_3_ core, and it was shown that the survival of the encapsulated phage increased by 38.1% compared to the native bacteriophage [12].

It is necessary to study the effect of polyelectrolytes on the preservation of their viability to encapsulate bacteriophages in PMC while maintaining their activity. At the moment, this problem has been little studied and has only a few publications, among which no works have been identified that study the effect of polyanions on the activity of bacteriophages. When creating polyelectrolyte microcapsules, biodegradable polyelectrolytes, such as dextran sulfate (DS), polylysine (PL), and polyarginine (PArg), are used as targeted delivery means. The work of Shima et al. [13] showed that the cationic polyelectrolyte poly-l-lysine decreased the bacteriophage activity 2.5 times at a polymer concentration of 10 μg/mL. At a concentration of 1 mg/mL, it shows complete inactivation of bacteriophage T4. In addition, there are polyelectrolytes derived from synthesized amino acids, such as poly-y-methyl-γ, L-glutamate, and poly-*N*-(p-aminoethyl)glutamine, which also reduce the activity of bacteriophages T4 and T5 [14]. In the work of Shalitin et al. [15], the suppression of phage activity is explained by the fact that the polyelectrolyte destroys the viral capsid, binds Phage DNA, and inactivates it. In this connection, before encapsulating the phage in the PMC, we studied the effect of polyelectrolytes on the infectious activity of coliprotein bacteriophage.

The aim of this work is to study the effect of polycations and polyanions on the activity of bacteriophages.

## 2. Materials and Methods

Dextran (D, MW 40,000) Sigma (Merck KGaA, Darmstadt, Germany), Poly-L-arginine hydrochloride (PArg, MW 15,000–70,000) Sigma (Merck KGaA, Darmstadt, Germany) and sodium dextran sulfate (DS, MW 15,000) Sigma (Merck KGaA, Darmstadt, Germany), polystyrenesulfonate sodium (PSS) and polyallylamine hydrochloride (PAH) with a molecular mass of 70 kDa, coliprotetic bacteriophage (Microgen NPO AO, Moscow, Russia), ethylenediaminetetraacetic acid (EDTA), calcium chloride (CaCl_2_ × 2H_2_O), sodium chloride and sodium carbonate from Reahim (Reahim AO, St.Petersburg, Russian Federation). Protease from Streptomyces griseus (Type VI No. P-5130) Sigma (Merck KGaA, Darmstadt, Germany).

### 2.1. Bacterial Strains and Their Cultivation

We used a daily culture of *Escherichia coli* K12. The strain was grown on agar LB medium in test tubes at 37 °C. A cell suspension was prepared in sterile distilled water, density 15 × 10^8^/mL (No. 5 according to McFarland).

### 2.2. Sowing

The suspension under study (phage with polyelectrolyte/native phage) in a volume of 100 μL and 100 μL of *E. coli* K12 suspension were applied to the 20 mL of a cooled molten medium of nutrient LB agar/MacConkey-GRM medium (Federal Budget Institution of Science «State Research Center for Applied Microbiology & Biotechnology», Obolensk, Russian Federation) in Petri dishes. After incubation for 2 days at 37 °C, the dishes were examined for the presence of lysis zones of the bacterial lawn. The presence of lysis zones of the bacterial lawn, plaques, indicated a viable lysogenic state of the bacteriophage. The sowing of each sample was carried out in triplicate.

## 3. Results

In this work, we studied the effect of positive and negative charged polyelectrolytes on the activity of coliprotetic bacteriophages.

**Figure 1:**
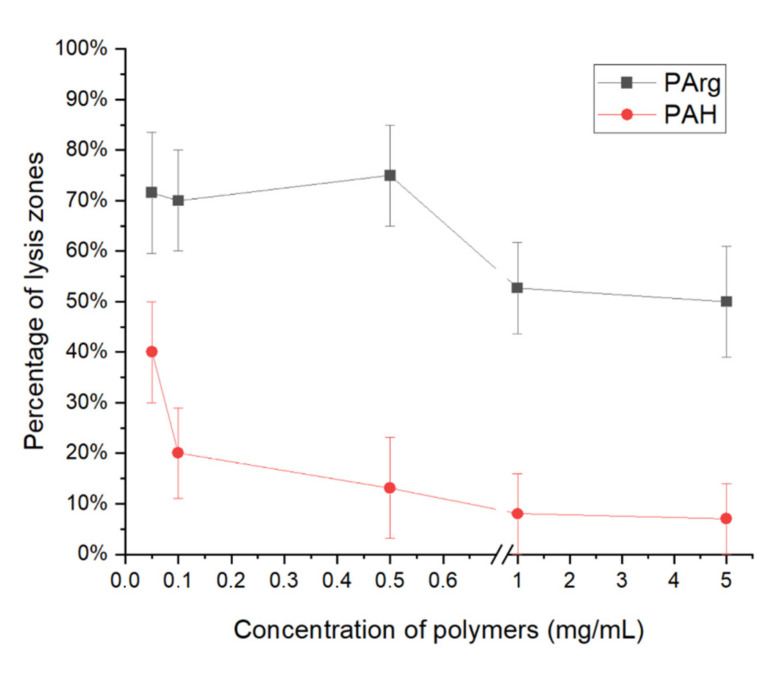
Plot of the percentage viability of bacteriophages against the concentration of positively charged polyelectrolytes polyallylamine and polyarginine of native bacteriophage.

At Figure 1 the viability of bacteriophages in the presence of positively charged polyelectrolytes polyallylamine (PAH) and polyarginine (PArg) was shown.

As can be seen from Figure 1, polyallylamine decreased the viability of bacteriophage during increasing polyelectrolyte concentration (from 0.05 mg/mL to 5 mg/mL). Presumably, PAH may inhibit phage activity due to the electrostatic nature of polyelectrolyte, which is oppositely charged to the bacteriophages capsid surface. That hypothesis is confirmed by the literature [13], in which polylysine has an antiphage activity and a structure similar to PAH.

Also, at Figure 1, polyarginine gradually inhibits the bacteriophage activity due to the concentration of polyelectrolyte from 0.05 to 5 mg/mL. Polyarginine has a lower inhibitory effect (then PAH) on the activity of the bacteriophage, and it may be related to the charged NH^+^ group of the polyelectrolyte (which provides interaction with the phage capsid) located near an uncharged NH_2_ group, which sterically prevents the polyelectrolyte from binding to the capsid.

The results of experiments on the interaction of polyallylamine with phage capsid in the presence of salts may be the confirmation of the electrostatic nature of its interaction. In the works of Tikhonenko et al. [16], the inactivating effect of this polyelectrolyte on the activity of enzymes was demonstrated, and it was also proposed to neutralize this effect in two ways. First, increasing the salt concentration in the solution, as a result of which the formation of polyelectrolyte-protein complexes weakens according to the classical law of statistical physics, which binds the Debye radius with the ionic strength of the solution. Alternatively, add up to 5 mM ammonium sulfate, which has a high ability to form strong bonds between PAH and the divalent ${\mathrm{SO}}_{4}^{2-}$ anion.

**Figure 2:**
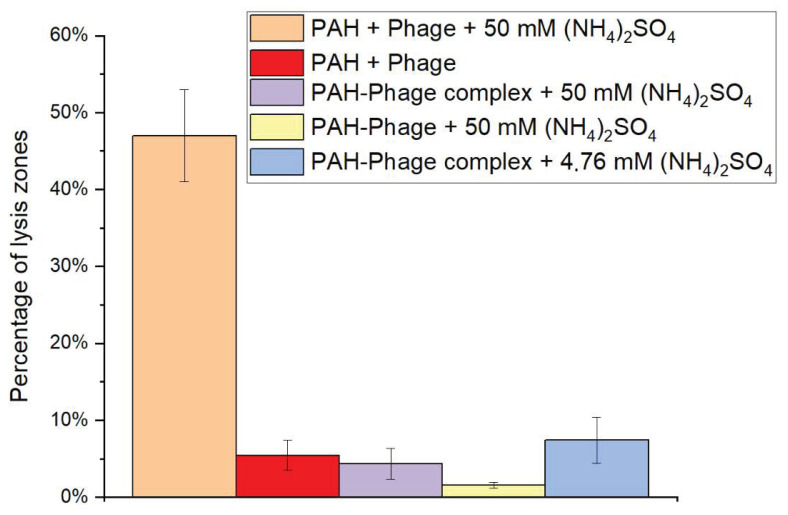
Influence of polyallylamine to bacteriophage in presence of (NH_4_)_2_SO_4_.

In this connection, we incubated polyallylamine with 50 and 4.76 mM ammonium sulfate, after which a suspension of bacteriophages was added. Figure 2 shows the influence of polyallylamine to bacteriophage in presence of (NH_4_)_2_SO_4_.

As can be seen from Figure 2, in the case of using 50 mM ammonium sulfate, the activity of the bacteriophage decreased by 47% compared to the native virus, although the activity of the phage incubated in pure PAH was almost absent. In the case of using 4.76 mM ammonium sulfate, almost complete inhibition of the bacteriophage was observed. Thus, we can conclude that inhibition of a bacteriophage is an electrostatic nature and the use of high ionic strength will prevent the formation of the PAH-protein capsid complex, which obeys the classical law of statistical physics, relating the Debye radius to the ionic strength of a solution.

The next step was to study the possibility of restoring the activity of bacteriophages, which were incubated for 10 min in a PAH solution, after which they were kept in a solution of ammonium sulfate 50 mM and with 4.76 mM. Figure 2 shows that ammonium sulfate in none of the concentrations restored the activity of the inhibited phage.

**Figure 3:**
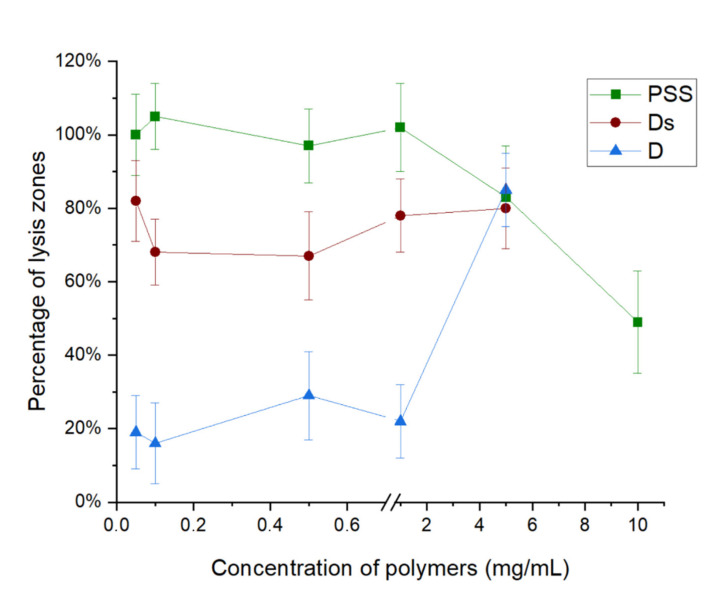
Plot of the percentage viability of bacteriophages against the concentration of polystyrene sulfonate (PSS), dextran (D), and dextran sulfate (DS) of native bacteriophage.

At Figure 3 the viability of bacteriophages in the presence of negatively charged polyelectrolytes polystyrene sulfonate (PSS), dextran sulfate (DS), and non-charged dextran (D)was shown.

As can be seen from Figure 3, polystyrene sulfonate does not affect the viability of bacteriophage during the increasing polyelectrolyte concentration from 0.05 mg/mL to 1 mg/mL. That effect relates to electrostatic repulsion of bacteriophage against polyelectrolyte such as both have a negative charge. PSS decrease the viability of bacteriophage from 5 mg/mL of polyelectrolyte concentration. The inhibition effect of polystyrene sulfonate effect may be related to the increasing of hydrophobic interaction with phage capsid. We suppose that hydrophobic interaction appears the cause of an excess of polyelectrolyte molecules, which can not to repulsed from capsid cause of limited numbers of negatively charged sites of the bacteriophage’s surface.

Also, at Figure 3 dextran sulfate inhibits bacteriophage activity at 20–30% was shown. We assume that DS partially inhibits bacteriophages cause of hydrophobic interaction with phage capsid, but complete inhibition is absent cause of the electrostatic partial repulsion of the polyelectrolyte against bacteriophage.

For confirmation of the hypothesis of hydrophobic inhibition, we researched the interaction between bacteriophage and dextran, which has almost the same structure as dextran sulfate, but it did not have a negative charge. Figure 3 shows that dextran inhibits bacteriophage activity by 80% at concentration diapason from 0.05 to 5 mg/mL due to hydrophobic interaction with phage capsid. Dextran lose its inhibition effect from a concentration of 5 mg/mL, related to conformation changing of dextran due to an increase in its concentration. The molecular dimensions of dextran decrease with increasing concentration [16], which can lead to a decrease in the interaction between the polymer and capsid.

We also studied the effect of polymers (polyarginine, polyallylamine, dextran sulfate, polystyrene sulfonate, dextran) on the survival of *E. coli*, and it was shown that these polyelectrolytes do not affect its viability.

## 4. Conclusions

The work studied the effect of polyallylamine, polyarginine, polystyrene sulfonate, dextran sulfate, and dextran on the activity of coliprotetic bacteriophages.

The study of positively charged polyelectrolytes polyallylamine and polyarginine showed that polyallylamine decreased viability of bacteriophage during increasing polyelectrolyte concentration (from 0.05 mg/mL to 5 mg/mL). Polyarginine gradually inhibits the bacteriophage activity due to the concentration of polyelectrolyte from 0.05 to 5 mg/mL. Polyarginine has a lower inhibitory effect (then polyallylamine) on the activity of the bacteriophage, it may be related to the charged NH^+^ group of the polyelectrolyte (which provides interaction with the phage capsid) located near an uncharged NH_2_ group, which sterically prevents the polyelectrolyte from binding to the capsid.

It was shown that the inhibition of the bacteriophage by polyallylamine has an electrostatic nature and the use of high ionic strength will prevent the formation of the PAH-protein capsid complex, which obeys the classical law of statistical physics, which relates the Debye radius to the ionic strength of a solution.

The effect of negatively charged polyelectrolytes polystyrene sulfonate, dextran sulfate, and dextran on bacteriophage viability was studied. Polystyrene sulfonate does not affect the viability of bacteriophage during increasing polyelectrolyte concentration from 0.05 mg/mL to 1 mg/mL. Polystyrene sulfonate decreases the viability of bacteriophage since 5 mg/mL of polyelectrolyte concentration, which is related to the appearance of hydrophobic interaction between capsid and polystyrene sulfonate. Dextran sulfate inhibits bacteriophage activity at 20–30% also related to hydrophobic interaction. Dextran inhibits bacteriophage activity by 80% at concentration diapason from 0.05 to 5 mg/mL due to hydrophobic interaction with phage capsid. That result confirms the hypothesis that DS partially inhibits bacteriophages by cause of hydrophobic interaction with phage capsid because dextran, which has almost the same structure as dextran sulfate but does not have a negative charge.
